# The amount of drunk days and its relationships with other characteristics of alcoholic behavior of the respondents

**DOI:** 10.1192/j.eurpsy.2024.847

**Published:** 2024-08-27

**Authors:** V. Kuzminov, I. Linskiy, O. Minko, M. Denisenko, T. Tkachenko, V. Zadorozhny, N. Malykhina, O. Minko, R. Lakinskiy, O. Vasilyeva, B. Herasymov, D. Herasymov, O. Yurchenko

**Affiliations:** ^1^Emergency psychiatry and narcologyand narcology, SI “Institute of Neurology, Psychiatry and Addiction of the NAMS of Ukraine”, Kharkiv, Ukraine

## Abstract

**Introduction:**

Alcohol abuse is a multifaceted problem. Its harmful effects on the individual have been more studied. Less studied is the influence of a drinking person on his microsocial environment. Having an abusing person in his space makes it interesting to study the influence of some quantitative and qualitative indicators of his alcohol consumption on the microsocial environment.

**Objectives:**

1532 people were examined during 2018-2022, who belonged to three comparison groups: patients with alcohol dependence (AD) (401 people); healthy relatives of AD patients (725 people); representatives of the general population comparable with the representatives of the first two age groups (406 people).

**Methods:**

The main research instruments were the questionnaire of the international research consortium “GENAHTO” (Gender, Alcohol, and Harms to Others), as well as the Alcohol Use Disorders Identification Test (AUDIT). The obtained data were processed by methods of mathematical statistics (variance, correlation and regression analysis).

**Results:**

An algorithm for regression analysis in conditions of high dispersion of the initial data has been developed. Using this algorithm, it was shown that the regression dependence of the main characteristics of alcohol behavior on the frequency of DD is non-linear, while for typical and maximum doses of alcohol it is optimally described by polynomials of the second degree, and for the severity of disorders due to alcohol use (AU), the time spent on AU, as well as self-assessment of the negative impact of AU by respondents on their environment - by polynomials of the third degree. It was found that for men (on average) to reach the border of risky-dangerous AU (according to the criteria of the AUDIT test), a lower frequency of DD is sufficient than for women, which indicates a greater vulnerability of men (compared to women) to the formation of disorders due to AU.

**Conclusions:**

It was established that for men (on average) to reach the limit of risky and dangerous (according to the criteria of the AUDIT test), a lower frequency (2-3 times a week) is enough than for women (3-4 times a week), which once again indicates greater vulnerability of men (compared to women) to the formation of due to psychological problems and mental disorders.

**Disclosure of Interest:**

None Declared

